# Editorial: Curricular proposals for physical education: local, global and transnational perspectives

**DOI:** 10.3389/fspor.2025.1574454

**Published:** 2025-02-27

**Authors:** Ingrid Wiggers, Allyson Carvalho de Araújo, Flavia Martinelli Ferreira, Juliana Freire

**Affiliations:** ^1^ Faculty of Physical Education, University of Brasília, Brasília, Brazil; ^2^ Department of Physical Education, Federal University of Rio Grande do Norte, Natal, Brazil; ^3^ Faculty of Physical Education, State University of Campinas, Campinas, Brazil; ^4^ Faculty of Education, University of Ottawa, Ottawa, ON, Canada

**Keywords:** physical education, curriculum, cross-border studies, comparative studies, knowledge

**Editorial on the Research Topic**
Curricular proposals for physical education: local, global and transnational perspectives

## Introduction

Historically, physical education (PE) has been understood as a subject centred on developing sports performance through specific skills and techniques or on promoting health through discourses such as “*fitness for health”* or “*fitness for sport”* (1). However, influenced by the principles of critical pedagogy, PE curricula across various countries have continued to evolve, with these ongoing developments still under analysis [e.g., (2, 3)].

Contemporary researchers examine how knowledge is organized within health and physical education curricula, and frequently employ cross-border (4) or comparative studies to foster knowledge exchange and broaden perspectives across diverse sociocultural contexts. By addressing specific themes such as health [e.g., (5)] or technology [e.g., (6, 7)], these studies provide valuable opportunities to contrast and enrich the field of physical education through different experiential contexts.

We argue that the curriculum should not be perceived merely as a static representation of educational policy—a prescriptive document for schools. Instead, it constitutes a dynamic and ongoing process of defining and representing the knowledge deemed essential for future generations while simultaneously adapting to evolving pedagogical practices (8). For this reason, we underscore the significance of comparative studies in advancing pedagogical practices within physical education [e.g., (9)].

Contemporary challenges prompt critical reflections on the curriculum, particularly regarding its social relevance. Comparative research offers a valuable avenue for knowledge exchange in addressing the pedagogical challenges associated with physical education curricula. While each country or region follows distinct policies, the similarities and differences across these contexts provide meaningful insights that contribute to professional reflection and foster academic dialogue at an international level.

This Research Topic brings together a collection of papers that explore diverse perspectives on physical education curricula. It presents a rich dialogue, featuring four articles that engage discussions across seven countries—Brazil, Uruguay, Spain, Colombia, Chile, Portugal, and Italy. Although each article focuses on its specific context, they collectively encourage readers to think beyond these realities, fostering broader reflections on curriculum development and pedagogical practices.

## Summary of selected articles from this research topic

Araújo et al. examine how digital media and technologies are incorporated into PE curricula in five Ibero-American countries (Brazil, Uruguay, Chile, Colombia, and Spain). Their study highlights the growing role of digital technologies in education, even in traditionally experiential subjects like PE. While all five countries recognize their relevance, the curricula differ: some treat media and technology as autonomous elements, while others integrate them across subjects like health and physical education (HPE).

The research identifies two main approaches: a critical perspective, which fosters analysis and engagement, and an instrumental approach, where technology is seen as a teaching tool. Advocating for a more integrated and dynamic use of digital media, the study calls for curricular reevaluations and further research on teachers' application of these policies in practice.

Souza et al. analyze the evolution of PE in Brazil, focusing on Ceará and Quixadá. They describe a shift over the past forty years from sport- and health-centred approaches to a broader focus on movement, the body, and cultural dynamics. This transition challenges the longstanding positivist paradigm, reflecting a deeper epistemological and methodological transformation in PE pedagogy.

The article examines how theoretical shifts in the PE curriculum, particularly the systematization proposed by a community of teacher-researchers, influence PE teachers' pedagogical practices in Quixadá. Using a collaborative action research approach—including classroom observations, group discussions, and field diaries—the study fosters dialogue between teachers and researchers, promoting reflection on curriculum and practice.

In the Italian context, Monacis et al. explore PE teachers' perceptions of the teaching styles they use in curricular lessons. The study examines how these styles impact students' engagement and learning in motor skills, fitness, and health. It finds that reproduction-based teaching styles are predominant, regardless of teachers' years of service. While offering valuable insights, the study leaves questions about the practical application of teaching styles in PE largely unexplored.

Onofre et al. provide a historical overview of the research of physical education and sports didactics in Portugal, tracing key trends and the shift toward more inclusive, student-centred approaches. Their forward-looking research agenda emphasizes interdisciplinary and international collaboration, calling for comparative studies both within Europe and between continents—aligning with the focus of this research topic.

## Invitation

The articles in this Research Topic of *Frontiers in Sport and Active Living* contribute to the expanding body of literature on cross-border and comparative studies. We invite scholars and practitioners to engage in this theoretical and methodological endeavour, fostering a deeper understanding by exploring both similarities and differences across our respective contexts.
